# Editorial: Highly contiguous plant genome assembly and transcriptional regulation

**DOI:** 10.3389/fpls.2023.1331498

**Published:** 2023-12-13

**Authors:** Xue-Chan Tian, Kai-Hua Jia

**Affiliations:** ^1^Institute of Crop Germplasm Resources, Shandong Academy of Agricultural Sciences, Jinan, China; ^2^National Engineering Research Center of Tree Breeding and Ecological Restoration, Beijing Advanced Innovation Center for Tree Breeding by Molecular Design, National Engineering Laboratory for Tree Breeding, Key Laboratory of Genetics and Breeding in Forest Trees and Ornamental Plants, Ministry of Education, College of Biological Sciences and Technology, Beijing Forestry University, Beijing, China

**Keywords:** genome assembly, Transcriptional regulation, secondary metabolites, TO-GCN, miRNA

## Introduction

The past decade has seen a paradigm shift in plant genomics, with the advent of novel sequencing technologies and bioinformatic tools transforming our understanding of genetic complexity and diversity. The plant genome highly influences its functional and regulatory mechanisms, which in turn determine the plant’s growth, development, and capacity to withstand environmental changes. Advances in long-read and high-fidelity sequencing technologies have revolutionized how plant genomes can be studied, enabling the generation of novel biologically accurate genomes. Understanding the complexity of the plant genome is vital, as it underpins the effective application of genetic and genomic resources to address challenges such as food security, climate change, and preserving biodiversity. In this context “*Highly Contiguous Plant Genome Assembly and Transcriptional Regulation*”, the Research Topic of this Research Topic, underscores the significance of advancing plant genomics research to navigate the challenges of biological and environmental change. The Research Topic presents an assembly of remarkable studies and insights about cutting-edge genome sequencing methods, high-resolution plant genome assembly and the complexity of plant transcriptional regulation.

## Advancements in highly contiguous plant genome assembly

This Research Topic exemplify the trend of integrating multiple sequencing technologies to achieve high-resolution plant genome assemblies. For instance, the *Quercus acutissima* (Liu et al.)*, Alpinia oxyphylla* (Pan et al.) and *Melastoma candidum* (Zhong et al.) genome were effectively assembled employing a combination of PacBio long read, Hi-C, and Illumina short read technologies. Likewise, the *Lindera megaphylla* genome (Tian et al.) was assembled through the utilization of ONT (Oxford Nanopore Technologies) long-read sequencing supplemented with Hi-C scaffolding technologies. The *Thalia dealbata* genome (Tang et al.) genome, a pioneering sequenced genome in the Marantaceae family, was assembled using PacBio HiFi reads and Hi-C technology. Additionally, a high-quality chromosome-scale reference genome of *Castanopsis hystrix* (Huang et al.) was obtained using a similar combination of Illumina and PacBio HiFi reads with Hi-C technology. Moreover, the innovative trio-binning method employed in the chili pepper genome assembly adroitly addressed the commonplace challenge of haplotype-switching associated with traditional plant genome assembly methods (Delorean et al.). The successful application of the trio-binning method underscores our evolving understanding and technological advancements in unraveling the complexities inherent in plant genomes.

## Functional genomic insights

Following genome assembly, the research featured in this Research Topic offers a comprehensive perspective on functional genomics across a range of plant species. In Lauraceae family, the species revealed unique gene duplications and microsynteny related to isoquinoline alkaloids, elucidating the genetic mechanisms of wood decay resistance. The genome of *Melastoma candidum* provided critical insights into trichome evolution, with whole genome duplications playing a significant role in the expansion of trichome-related genes, thereby highlighting the impact of genomic alterations in morphological diversity. The exploration of *Alpinia oxyphylla* delved into genomic, transcriptomic, and metabolic profiles, correlating genomic variations with the synthesis of pharmacodynamic compounds like nootkatone. *Castanopsis hystrix* exhibited gene family expansions and contractions, pivotal for adapting to tropical and subtropical climates, enriching our understanding of the genomic foundations of environmental adaptation in forest trees. The genome of *Thalia dealbata*, a notable wetland plant, contributed substantially to the understanding of evolutionary adaptations to wetland environments and phylogenomic research in Marantaceae and Zingiberales.

## Unraveling the complexity of plant transcriptional regulation

Another prominent theme across these studies is the intricate nature of plant transcriptional regulation. In *Epimedium pubescens*, Xu et al. employed high-temporal-resolution transcriptome analysis coupled with metabolite profiling, uncovering the biosynthesis pathways of bioactive compounds. Chen et al. employed a miRNA-TF-mRNA network to investigate wood development in rubber trees, shedding light on phenylpropanoid and lignin biosynthesis, thus enriching our knowledge of molecular regulatory mechanisms. Likewise, Cao et al.‘s investigation of secondary trunk development in *Ginkgo biloba* identified key regulatory pathways and contributed further to our understanding of evolutionary and developmental biology in the context of plant genomics. By presenting these distinct yet connected studies, this Research Topic contributes to expanding our understanding of the vast and intricate world of plant transcriptional regulation.

## Conclusion

The articles presented in this Research Topic contribute significantly to our understanding of highly contiguous plant genome assembly and transcriptional regulation. The genome assembly studies provide valuable resources for further ecological and evolutionary studies in *Alpinia oxyphylla, Capsicum annuum, Castanopsis hystrix, Ginkgo biloba*, *Lindera megaphylla, Melastoma candidum*, *Quercus acutissima* and *Thalia dealbata*. The gene regulation studies shed light on the molecular mechanisms underlying the biosynthesis of phenolic and flavonoid glycosides, trichome color variation, and secondary trunk development. These findings have implications for plant adaptation, diversification, and ecological interactions. Overall, this Research Topic highlights the importance of comprehensive genomic studies in unraveling the complexities of plant biology and provides a foundation for future research in this field.

## Author contributions

X-CT: Writing – original draft, Writing – review & editing. K-HJ: Writing – original draft, Writing – review & editing.

